# Brain activation during dual-task processing is associated with cardiorespiratory fitness and performance in older adults

**DOI:** 10.3389/fnagi.2015.00154

**Published:** 2015-08-12

**Authors:** Chelsea N. Wong, Laura Chaddock-Heyman, Michelle W. Voss, Agnieszka Z. Burzynska, Chandramallika Basak, Kirk I. Erickson, Ruchika S. Prakash, Amanda N. Szabo-Reed, Siobhan M. Phillips, Thomas Wojcicki, Emily L. Mailey, Edward McAuley, Arthur F. Kramer

**Affiliations:** ^1^Neuroscience Program, University of Illinois at Urbana-ChampaignUrbana, IL, USA; ^2^The Beckman Institute for Advanced Science and Technology, University of Illinois at Urbana-ChampaignUrbana, IL, USA; ^3^Department of Psychology, University of IowaIowa City, IA, USA; ^4^The Center for Vital Longevity, School of Behavioral and Brain Sciences, University of Texas at DallasDallas, TX, USA; ^5^Department of Psychology, University of PittsburghPittsburgh, PA, USA; ^6^Department of Psychology, The Ohio State UniversityColumbus, OH, USA; ^7^Cardiovascular Research Institute, University of Kansas Medical CenterKansas City, KS, USA; ^8^Department of Preventative Medicine, Northwestern University Medical SchoolChicago, IL, USA; ^9^Exercise Science, Lansing School of Nursing and Health Sciences, Bellarmine UniversityLouisville, KY, USA; ^10^Department of Kinesiology, Kansas State UniversityManhattan, KS, USA; ^11^Department of Kinesiology and Community Health, University of Illinois at Urbana-ChampaignUrbana, IL, USA

**Keywords:** exercise, aging, fMRI, dual-task, cardiorespiratory fitness, executive function

## Abstract

Higher cardiorespiratory fitness is associated with better cognitive performance and enhanced brain activation. Yet, the extent to which cardiorespiratory fitness-related brain activation is associated with better cognitive performance is not well understood. In this cross-sectional study, we examined whether the association between cardiorespiratory fitness and executive function was mediated by greater prefrontal cortex activation in healthy older adults. Brain activation was measured during dual-task performance with functional magnetic resonance imaging in a sample of 128 healthy older adults (59–80 years). Higher cardiorespiratory fitness was associated with greater activation during dual-task processing in several brain areas including the anterior cingulate and supplementary motor cortex (ACC/SMA), thalamus and basal ganglia, right motor/somatosensory cortex and middle frontal gyrus, and left somatosensory cortex, controlling for age, sex, education, and gray matter volume. Of these regions, greater ACC/SMA activation mediated the association between cardiorespiratory fitness and dual-task performance. We provide novel evidence that cardiorespiratory fitness may support cognitive performance by facilitating brain activation in a core region critical for executive function.

## Introduction

Successful and healthy aging is an important public health priority as the population of adults 65 and older is projected to almost double from 43.1 million in 2012 to 83.7 million in 2050 (Ortman et al., 2014). The rapid growth of the older adult population is significant as aging is associated with decline in both physical and cognitive health. Physical decline involves increased frailty, arthritis, and incidence of chronic diseases such as cardiovascular disease, type 2 diabetes, and cancer (Fried, 2000). Cognitive aging is most frequently found in measures of processing speed, executive function, and memory (Park and Reuter-Lorenz, 2009; Salthouse, 2010).

Importantly, physical and cognitive health is strongly associated with physical activity and sedentary behaviors. With advancing age, older people tend to be less active, engage in less strenuous physical activity, and spend more time sitting (Evenson et al., 2011). Sedentariness is a risk factor for diabetes (Thorp et al., 2011), depression (de Wit et al., 2011), has negative impact on brain health (Voss et al., 2014), and may accelerate physical decline (Booth et al., 2011). Exercise is a promising lifestyle factor that may combat the negative effects of both physical and cognitive aging by promoting broad positive physiological effects, as well as reducing the risk of cardiovascular and other chronic diseases (Kujala, 2009) and increasing brain and cognitive health (Colcombe and Kramer, 2003). Thus, examining lifestyle factors and interventions that have the potential to reduce or reverse age-related decline has important implications for the expanding older adult population.

Specific measures of brain health are associated with cardiorespiratory fitness. Older adults with higher cardiorespiratory fitness show reduced incidence of cognitive decline (Barnes et al., 2003). Cardiorespiratory fitness is also associated with less structural brain atrophy (Colcombe et al., 2003; Erickson et al., 2009), enhanced brain function (Colcombe et al., 2004; Voss et al., 2010; Prakash et al., 2011), and higher estimates of white matter integrity (Marks et al., 2011; Johnson et al., 2012; Burzynska et al., 2014).

Additionally, cardiorespiratory fitness in healthy older adults has been linked to better cognitive performance in processes especially vulnerable to age, such as executive function (Colcombe and Kramer, 2003; Colcombe et al., 2004; Smith et al., 2010; Guiney and Machado, 2013). Executive function is required to direct behavior through implementing cognitive control and can be decomposed into processes of shifting, updating, and inhibition (Miyake et al., 2000). Although executive function tasks recruit a distributed network of brain regions that span both frontal and posterior parietal areas (Collette et al., 2006; Niendam et al., 2012), the prefrontal cortex (PFC) acts to actively maintain and manipulate goal-directed representations (Miller and Cohen, 2001). Various tasks can be used to assess executive function and one specific example is the dual-task paradigm, as it requires coordination, maintenance, and integration of two tasks. Studies have found that the dual-task paradigm is sensitive to both age (Verhaeghen et al., 2003) and exercise (Hawkins et al., 1992) effects. A meta-analysis of 33 studies reported that both younger and older adults experience performance decrements when performing simultaneous tasks; however, older adults demonstrated a greater dual-task performance deficit than younger adults (Verhaeghen et al., 2003). Importantly, Hawkins et al. (1992) demonstrated that 10-weeks of aquatic exercise resulted in improved dual-task performance in older adults compared to the non-exercise control group.

Beyond behavioral performance, cardiorespiratory fitness and physical activity are associated with differential brain activation in older adults. Enhanced activation in areas involved in executive function, such as the PFC and parietal cortex, were coupled with better performance on executive function tasks (e.g., flanker task, Stroop task, and digit symbol substitution task; Colcombe et al., 2004; Rosano et al., 2010; Prakash et al., 2011). However, no studies have specifically examined the extent to which greater cardiorespiratory fitness is related to greater *dual-task processing* in areas involved in executive function.

Thus, here we extend previous work by determining whether individual differences in cardiorespiratory fitness are associated with brain function in regions known to support executive function, particularly dual-task performance, in healthy older adults. Other studies report that brain volume (Erickson et al., 2009; Verstynen et al., 2012; Weinstein et al., 2012), resting state functional connectivity (Voss et al., 2010), and neuronal metabolites (Erickson et al., 2012) mediate the association between cardiorespiratory fitness and cognitive performance. Based on this previous literature, we hypothesized that older adults with greater cardiorespiratory fitness would exhibit greater PFC activation during dual-task processing and better dual-task performance. Furthermore, we examined whether brain activation during dual-task processing within the PFC mediated the association between cardiorespiratory fitness and cognitive performance.

## Materials and Methods

### Participants

Participants were recruited from the local community of Urbana–Champaign, IL, USA. Eligible participants met the following criteria: (1) 55–80 years of age, (2) a score ≥51 out of 57 on the modified Mini-Mental Status Exam (mMMSE, Stern et al., 1987), indicating absence of cognitive impairment that would impair every day function, but we acknowledge that the mMMSE is not equivalent to a full clinical adjudication of cognitive impairment or dementia, (3) right handedness, as defined by at least a 75% on the Edinburgh Handedness Questionnaire (Oldfield, 1971), (4) a score <3 on the Geriatric Depression Scale (Yesavage et al., 1983; Shiekh and Yesavage, 1986), (5) normal color vision and a visual acuity of at least 20/40, (6) informed consent, as approved by the University of Illinois Institutional Review Board. Participants were also required to meet magnetic resonance imaging (MRI) safety criteria, including no history of head trauma, head or neck surgery, diabetes, neuropsychiatric or neurological conditions (e.g., claustrophobia or brain tumors), as well as not having any ferrous metallic implants that could cause injury due to the magnetic field. All participants were compensated for their participation. Our participant sample consisted of 128 older adults.

### Cardiorespiratory Fitness Assessment

Consent from a personal physician was required before participants could engage in the cardiorespiratory fitness assessment. Graded maximal exercise testing on a motor-driven treadmill was used to assess cardiorespiratory fitness (VO_2_ max). To begin the test, the participant walked slightly faster than their normal walking pace (∼30–100 m/min) and every 2 min the grade increased by 2%. Medical personnel continuously monitored the participant’s oxygen uptake, heart rate, and blood pressure. Before the treadmill test, resting heart rate was measured while the participant was in a supine position after electrocardiogram preparation. Oxygen uptake (VO_2_) was measured from expired air samples taken at 30 s intervals until maximal VO_2_ (VO_2_ max) was attained or to the point of test termination due to symptom limitation and/or volitional exhaustion. VO_2_ max was defined was the highest recorded VO_2_ measurement when at least two of the three criteria were met: (1) plateau in VO_2_ peak between two or more workloads, (2) respiratory exchange ratio of greater than 1.00, and (3) a heart rate equivalent to their predicted maximum (i.e., 220 – age).

### Imaging Methods

**FIGURE 1:**
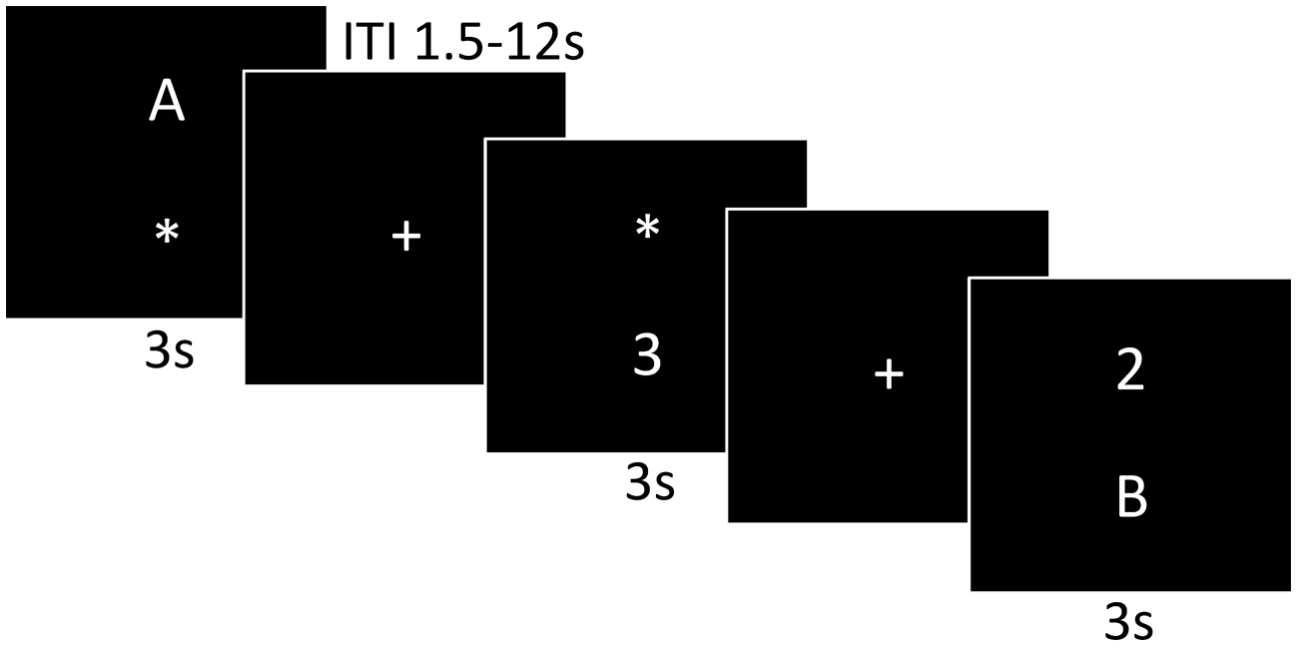
**A graphic illustration of the dual-task paradigm**. The first and third panels are examples of the single-task presentation, a letter or number discrimination, respectively. The last panel represents the dual-task presentation consisting of both the number and letter discrimination.

Functional MRI (fMRI) scans were acquired during an event-related dual-task paradigm. The dual-task is a task that provides a measure of executive function that requires maintenance and coordination of two tasks as well as inhibition of inappropriate responses. As shown in **Figure 1**, the dual-task paradigm consisted of two discrimination tasks and two conditions. The letter discrimination task was discrimination between “A” or “B” and the number discrimination task was discrimination between “2” or “3.” The single-task condition presented either only the letter or only the number discrimination task and the dual-task condition presented the letter and number discrimination tasks simultaneously. There were 48 single-task condition trials (24 letter and 24 number) and 48 dual-task condition trials, for a total of 96 trials. To respond, participants used their right index finger for “A” and right middle finger “B” and their left index finger for “2” and left middle finger for “3.” The number and letter stimuli were randomly presented above or below the location of the prior central fixation cross. When only a single stimulus was presented an “∗” held the place of the second stimulus to display a constant number of visual stimuli across condition types. Each trial began with a 1.5 s fixation cross followed by 3 s stimulus presentation, within which the participant responded. To optimize stimulus sequence and timing the inter-trial interval (ITI) ranged from 1.5 to 12 s with a mean ITI of 3.3 s. The participants were instructed to respond to the stimuli as fast and as accurately as possible with the button press on the MR-compatible response pad and the task stimuli were presented with MRI-safe fiber optic goggles (Resonance Technologies, Inc.). Brief practice on the task was conducted before the participant entered into the MR scanner to orient the participant to the task.

The conditions, trial types, and location of stimuli were randomized and first order counterbalanced. Performance measures of median response time and error rate were collected for the single-task and dual-task condition types. Median response time was used to reduce the influence of outliers. To reduce the dimensionality of the performance measures, a summed *z*-score of response time and error rate for each condition, Zsingle and Zdual, were calculated and used as the primary performance outcomes.

During the task, T2^∗^ weighted images were acquired using a fast echo-planar imaging (EPI) sequence with blood oxygenation level dependent (BOLD) contrast (64 × 64 matrix, 4 mm slice thickness, TR = 1500 ms, TE = 26 ms, flip angle = 60). A total of 414 volumes were acquired per participant (∼4.6 min).

Additionally, for all participants a high resolution T1-weighted structural brain image was acquired with a 3D (Magnetization Prepared Rapid Gradient Echo Imaging (MPRAGE) protocol with 114 contiguous axial slices, collected in ascending fashion parallel to the anterior and posterior commissures, echo time (TE) = 3.87 ms, repetition time (TR) = 1800 ms, field of view (FOV) = 256 mm, acquisition matrix 192 mm × 192 mm, slice thickness = 1.3 mm, and flip angle = 8°. All images were collected on a 3T head-only Siemens Allegra MRI scanner.

### Image Analysis

#### fMRI Preprocessing

FSL 5.0.4. (FMRIB’s Software Library^1^) was used for fMRI data preprocessing. MELODIC 3.13 (Multivariate Exploratory Linear Optimized Decomposition into Independent Components) and FIX 1.05 (FMRIB’s ICA-based Xnoiseifier) were used to reduce data artifacts (Smith et al., 2004; Salimi-Khorshidi et al., 2014). Within the MELODIC analysis, the following preprocessing steps were conducted: rigid body motion correction using MCFLIRT (Jenkinson et al., 2002), removal of non-brain structures using the brain extraction technique (BET; Smith, 2002), and temporal filtering with a high pass frequency cut off of 90 s. FIX was used as a semi-automatic ICA classification analysis and a representative subsample of 25 participants were used create a FIX training dataset. The subsample did not differ in age, sex, education, or mMMSE from the rest of the sample. The ICA components of the subsample were visually inspected and classified as ‘noise’ or ‘signal’ according to criteria explained in Kelly et al. (2010). The FIX training dataset was then applied to the remaining participants to reduce data artifacts. The ‘cleaned’ data was then spatially smoothed with a 6.0-mm full-width at half-maximum (FWHM) Gaussian kernel.

#### Registration

For each participant a two-step registration process was used to register all imaging data to the standard stereotaxic space of the 152T1 Montreal Neurological Institute (MNI) template. The first step was the registration between each participant’s low-resolution EPI image and the high-resolution T1 structural image. The second step was the registration between the participant’s high-resolution T1 structural image and the study specific template in the standard stereotaxic space of the 152T1 MNI template. The study-specific template was created using the representative subsample of the 25 subjects that were used to create the FIX training dataset. To create the study specific template each participant’s high-resolution T1 structural image was warped to standard stereotaxic space of the 152T1 MNI template via FLIRT, then an average of the registered high-resolution T1 images was created and spatially smoothed with a 6.0-mm FWHM Gaussian kernel. The study template was used to minimize the amount of warping during registration, to protect against registration bias, and to avoid registration complications in registering older adults to the MNI template. The registration process used FLIRT 12-parameter affine linear registration (Jenkinson and Smith, 2001; Jenkinson et al., 2002).

#### General Linear Model Analysis

After completing preprocessing and registration, the data were entered into the individual lower-level analysis with FEAT 6.00 (FMRI Expert Analysis Tool^2^) to measure brain activation during dual-task processing. The trial onsets of the single-task and dual-task trials were convolved with a double gamma hemodynamic response function (HRF) to create the distinct predictor models of the single-task and dual-task conditions. Additionally, error trials were modeled as a separate predictor of no interest. Four linear contrasts were produced: (1) single-task activation greater than fixation baseline, (2) dual-task activation greater than fixation baseline, (3) single-task activation greater than dual-task activation (single > dual), and (4) dual-task activation greater than single-task activation (dual > single). The contrasts generated four lower-level statistical parametric maps thresholded with a *z*-score of 2.33 and cluster threshold of *p* < 0.05. Based on our hypothesis that the dual-task will invoke differential brain activity compared to the single-task, the fourth contrast of dual > single was our contrast of interest.

Next, the four individual lower-level statistical parametric maps were used as inputs for a higher-level mixed-effects whole brain group analyses using FLAME (within FEAT; Beckmann et al., 2003). Within the regression analysis, cardiorespiratory fitness was used as our predictor of interest to examine where brain activation associated with dual-task processing was associated with individual differences of cardiorespiratory fitness. All group maps were thresholded with a *z*-score of 2.56 and cluster threshold of *p* < 0.05. Additionally age, sex, education (as these variables have been found to relate to cardiorespiratory fitness), and a voxel-wise gray matter partial volume images were included as covariates of no interest in the model. The gray matter partial volume images were used to ensure that individual differences in gray matter volume did not confound the results. The gray matter partial volume images were created by segmenting the MPRAGE image into gray, white, and CSF values using FSL’s automated segmentation technique (FAST) and then smoothing these images with a 6.0-mm FWHM kernel. The resulting clusters from the whole brain fMRI group analysis were used as regions of interest (ROI) in the subsequent analyses. In a secondary analysis of these ROIs, we explored the possibility that the activation patterns mediated the association between cardiorespiratory fitness and dual-task performance.

### Statistical Analysis

A repeated measures ANOVA was used to examine differences in response times and error rates between the single-task and dual-task conditions. To investigate the relationship between brain activation during dual-task processing and dual-task performance we conducted hierarchical multiple linear regressions. Analyses included age, sex, and education as covariates, given significant correlations between cardiorespiratory fitness and age, sex, and education. For all regression analyses, standardized *β*-values and *p*-values are reported as well as *t*-values when relevant.

A secondary mediation analysis was conducted to examine whether brain activation during dual-task processing mediated the association between cardiorespiratory fitness and dual-task performance. A mediation analysis is a hypothesis-driven model that describes a proposed mediating variable (M: brain activation during dual-task processing) that indirectly associates the independent variable (IV: cardiorespiratory fitness) and dependent variable (DV: dual-task performance). In the model, the coefficients of *a* and *b* represent the relationship between the IV and M, and the M and DV, respectively. It is now accepted that mediation analyses do not require an initial association between the IV and DV (Gelfand et al., 2009; Zhao et al., 2010; Rucker et al., 2011). Bootstrapping procedures were used to calculate the indirect effect to minimize problems attributed to a modest sample size (cf. MacKinnon, 2000; MacKinnon et al., 2004). Additionally, bootstrapping statistics make no *a priori* assumptions about the distribution of the paths *a*, *b*, and their product *ab* and demonstrate increased power without increasing Type 1 error rate (indirect effect; Preacher and Hayes, 2008a,b). The analysis was implemented using the PROCESS macro for SPSS developed by Preacher and Hayes^3^. Briefly, an empirical estimation of the sampling distribution of the product of the *a* and *b* path (*ab^∗^*) was generated by taking a new sample of size n with replacement from the available sample. Then, the *a* and *b* estimates were used to calculate *ab^∗^*, the indirect effect of cardiorespiratory fitness on dual-task performance, in a single resample of size n from the original data. This process was repeated *k* times (i.e., *k* = 10,000 bootstrap resamples). The distribution of *ab^∗^* served as an empirical non-parametric approximation of the sampling distribution of the indirect effect. The 95% bias corrected and accelerated confidence interval (corrected for both median bias and skew; Efron, 1987) of the indirect effect are derived by sorting the *ab^∗^* values from low to high and defining the lower [0.5 × (1 - 0.95) × 10,000 = 250th value in the sorted *ab^∗^* distribution] and upper [1 + 0.5 × (1 + 0.95) × 10,000 = 9,751st value] bounds. The null hypothesis of no indirect effect was tested by determining whether zero was within the confidence interval. If this is not the case, then there is evidence for a significant indirect effect. All models controlled for age, sex, and education. An alpha level of *p* < 0.05 and the 95% confidence interval of the indirect effect was used to determine significant effects. All behavioral data were analyzed with SPSS21.0 for Mac Computer.

## Results

Participant demographics and cardiorespiratory fitness data are presented in **Table 1**. Cardiorespiratory fitness was associated with age (*r* = -0.33, *p* < 0.05), sex (*r_pb_* = -0.51, *p* < 0.05) and education (*r* = 0.31, *p* < 0.05); these variables were thus used as covariates in all analyses.

**Table 1:** Demographics.

Demographic	Mean (SD)	Range
Age (years)	66.11 (5.54)	59–80
Education (years)	15.96 (2.93)	8–16
Sex (% Female)	67.2%	–
mMMSE	55.18 (1.61)	51–57
VO_2_max (mL/kg/min)	21.26 (4.77)	12.9–34.7

A repeated measures ANOVA was used to investigate performance differences between the single-task and dual-task. The main effect of condition for response time was significant [*F*(1,124) = 7.41, *p* < 0.05] suggesting the dual-task generated slower a response time (median = 1.95 s, SD = 0.23) compared to the single-task (median = 1.16 s, SD = 0.16). However, the main effect of condition for error rate [*F*(1,124) = 0.854, *p* > 0.05] was not significant.

Additionally, linear regressions were used to relate response time and error rate within each condition. There was a significant positive relationship between response time and error rate for both the single-task [β = 0.26, *t*(123) = 3.03, *p* < 0.01] and dual-task [β = 0.56, *t*(123) = 7.39, *p* < 0.01] while controlling for age, sex, and education. These results supported our reasoning for creating a combined *z*-score for response time and error rate for each condition because participants with shorter response time also exhibited lower error rates, or better overall performance.

### Cardiorespiratory Fitness and Dual-Task Performance

Higher cardiorespiratory fitness was associated with better performance only on the more difficult dual-task condition [Zdual: β = -0.25, *t*(126) = -2.9, *p* < 0.01], not the single-task condition [Zsingle: β = -0.16, *t*(126) = -1.76, *p* > 0.05]. The association between cardiorespiratory fitness and dual-task performance did not remain significant when controlling for age, sex, and education [β = -0.13, *t*(123) = -1.2, *p* > 0.05]. Our sample represents healthy, but low active older adults. Thus, our analysis may be underpowered to detect an association between cardiorespiratory fitness and dual-task performance while controlling for our covariates, moreover, this does not prevent further investigation with a mediation model (Hawkins et al., 1992; Rucker et al., 2011).

### Cardiorespiratory Fitness and Brain Activation

As predicted, the whole brain group analysis revealed that our main contrast of interest dual > single produced four clusters that were associated with individual differences in cardiorespiratory fitness (see **Table 2**). The four clusters were used as ROIs in our subsequent hierarchical multiple linear regressions and mediation analyses. Brain activation related to dual-task processing was represented as a subtraction of dual-task percent signal change minus single-task percent signal change. The whole brain analysis also indicated that the opposite contrast, single > dual did not result in any association between cardiorespiratory fitness and brain activation.

**Table 2:** Statistical peaks for brain activation patterns associated with higher levels of cardiorespiratory fitness and dual-task processing.

			Montreal Neurological Institute (MNI) peak coordinates		
Region	Cluster Size (#voxels)	Max *Z*	*X* (mm)	*Y* (mm)	*Z* (mm)
Anterior cingulate and supplementary motor cortex (ACC/SMA)	541	3.81	6	-2	50
Thalamus/Basal ganglia	757	3.57	-12	-18	10
Right motor/somatosensory cortex and MFG	406	3.99	50	-18	56
Left somatosensory cortex	331	4.78	-46	-24	56

**FIGURE 2:**
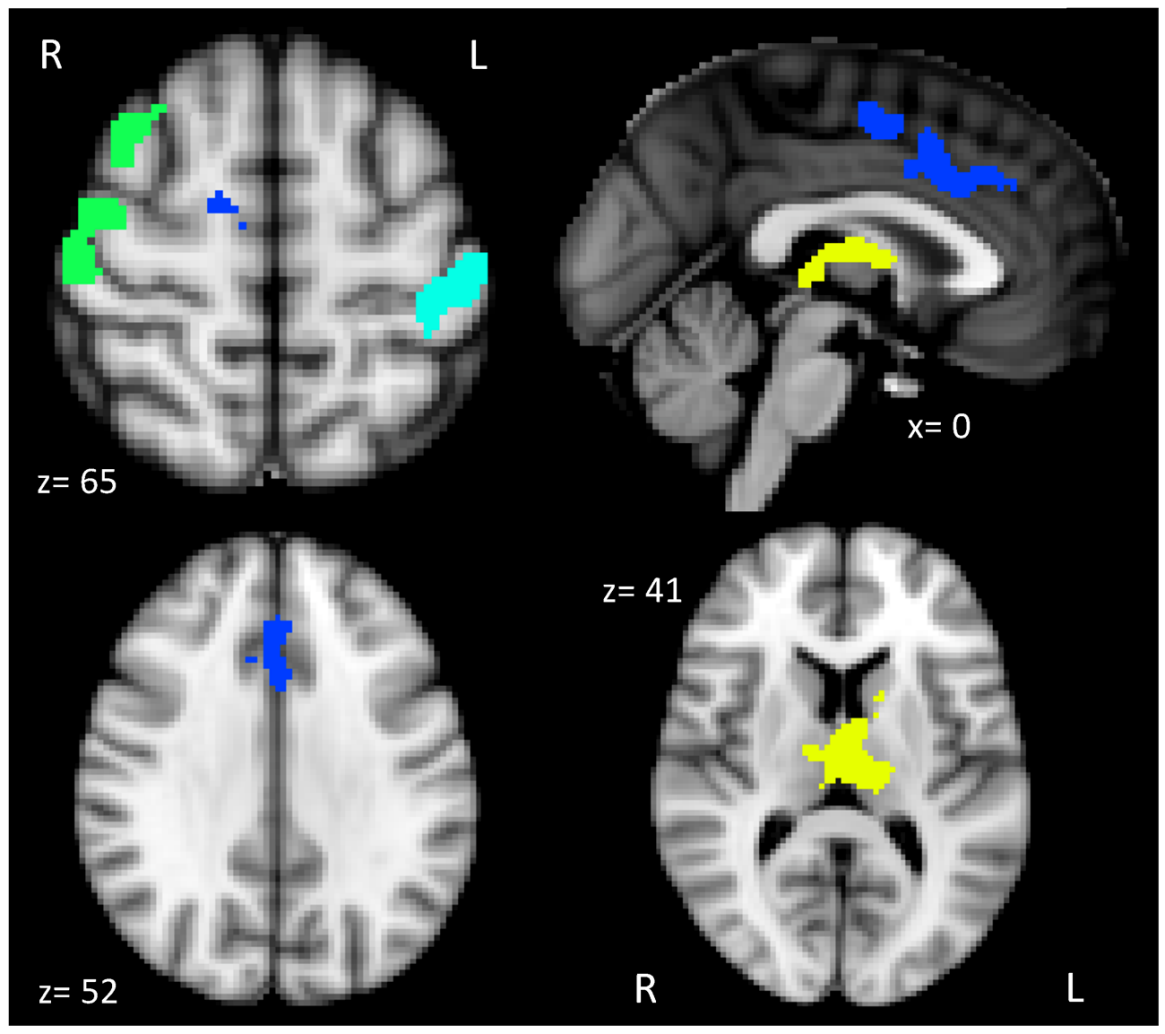
**Brain activation patterns associated with higher levels of cardiorespiratory fitness and dual-task processing**. The brain figure shows the four clusters of activation anterior cingulate and supplementary motor cortex (ACC and SMA; blue), thalamus and basal ganglia (yellow), right motor/somatosensory cortex and middle frontal gyrus (MFG; green), and left somatosensory cortex (teal).

**Figure 2** illustrates the four distinct clusters of activation that were positively associated with cardiorespiratory fitness, which included the anterior cingulate cortex (ACC) and supplementary motor cortex (SMA), thalamus and basal ganglia, right motor/somatosensory cortex and middle frontal gyrus (MFG), and left somatosensory cortex.

### Brain Activation and Dual-task Performance

**FIGURE 3:**
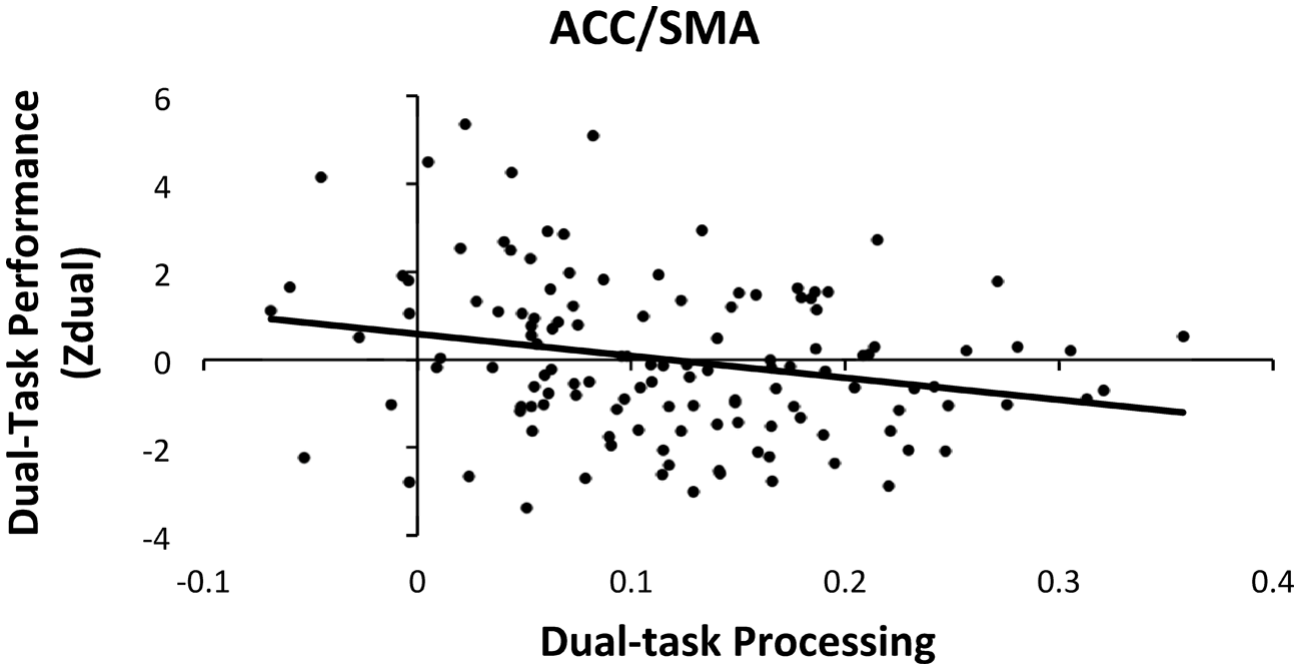
**Scatter plot of dual-task processing and performance (raw data)**. The plot illustrates the significant association between dual-task processing within the ACC/SMA and better dual-task performance (negative Zdual indicates faster RT and lower error rates).

Hierarchical multiple linear regressions were used to investigate the associations between dual-task performance and brain activation during dual-task processing in the four ROIs (ACC/SMA, thalamus/basal ganglia, right motor/somatosensory cortex and MFG, and left somatosensory cortex), controlling for age, sex, and education. To predict dual-task performance, the covariates (age, sex, and education) were entered as the first step and the four dual-task processing ROIs were entered as the second step. Both steps were significant [Step1: *F*(3,127) = 5.04, *p* < 0.05; Step 2: *F*(7,127) = 3.56, *p* < 0.05]. The addition of the four ROIs from the first to second step, resulted in a marginal increase of 6.3% of explained variance within dual-task performance [Δ*R*^2^ = 0.063, Δ*F*(4,120) = 2.23, *p* = 0.06]. The analysis also revealed that only the ACC/SMA ROI was significantly related with dual-task performance [β = -0.218, *t*(120) = -2.03, *p* < 0.05], which suggests that greater activation in the ACC/SMA had the strongest association with better dual-task performance as shown in **Figure 3**.

Therefore, an additional hierarchical multiple regression was conducted to discover the additional variance that the ACC/SMA ROI explained beyond the covariates and other three ROIs. The first step consisted of the covariates and the three ROIs unrelated to performance and the second step included the ACC/SMA ROI into the model. Both steps were significant [Step1: *F*(6,127) = 3.38, *p* < 0.05; Step 2: *F*(7,127) = 3.56, *p* < 0.05]. With the addition of the ACC/SMA ROI to the model, there was a significant increase of 2.9% of explained variance in dual-task performance [Δ*R*^2^ = 0.029, Δ*F*(1,120) = 4.13, *p* < 0.05].

### Mediation Analysis

**FIGURE 4:**
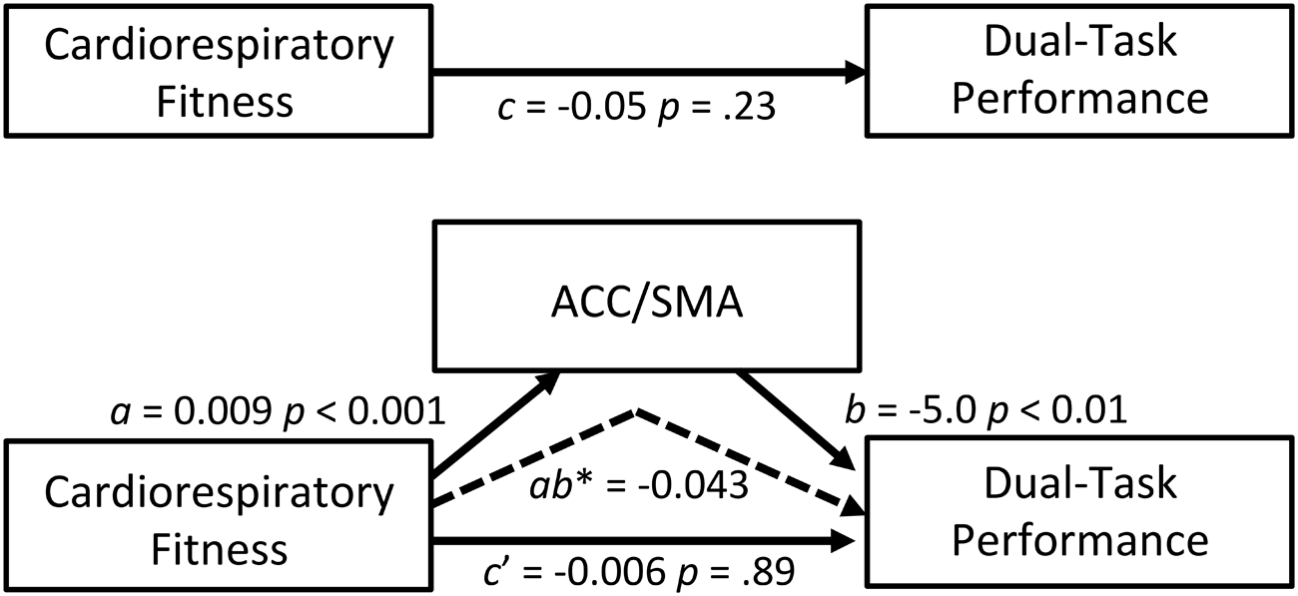
**Mediation model**. ACC/SMA as a mediator of the effect of cardiorespiratory fitness on dual-task performance. The 95% bootstrap confidence intervals of the indirect effect: -0.082, -0.012. The confidence intervals did not include zero, indicating reliable mediations (dashed arrow). Coefficients for all paths are unstandardized. *c* path: total (direct) effect of independent on dependent variable; *a* path: effect of independent on the mediator variable; *b* path: effect of the mediator on dependent variable; *c*′ path: indirect effect of independent on dependent variable through a mediator. *ab*^∗^: point estimate of the indirect effect of independent on dependent variable through a mediator (i.e., the multiplication of the paths *a* and *b*).

A mediation analysis was conducted as a secondary analysis to explore whether brain activation during dual-task processing mediated the association between cardiorespiratory fitness and dual-task performance. Of the four ROIs investigated, the ACC/SMA ROI emerged as a possible mediator because it was related to both cardiorespiratory fitness and dual-task performance. We acknowledge that the total effect between cardiorespiratory fitness and dual-task performance was not statistically significant after controlling for age, sex, and education; however, this is not a requirement to test mediation (Zhao et al., 2010; Rucker et al., 2011). Our mediation model was guided by theory with evidence that supports an association between exercise and dual-task performance (Hawkins et al., 1992). Thus, the model specifically addressed whether greater ACC/SMA activation (M) mediated the relationship between higher cardiorespiratory fitness (IV) and better dual-task performance (DV), while controlling for age, sex, and education. The ACC/SMA ROI significantly mediated the association between cardiorespiratory fitness and dual-task performance (indirect effect = -0.043; 95% CI = -0.082 to -0.012). **Figure 4** summarizes the coefficients, point estimates, and 95% bias-corrected and accelerated bootstrap confidence intervals of the mediation model. These findings support that greater activation within the ACC/SMA help to facilitate the relationship between cardiorespiratory fitness and better cognitive performance.

## Discussion

Higher levels of cardiorespiratory fitness have been associated with enhanced task-related brain activation in older adults (Colcombe et al., 2004; Prakash et al., 2011). However, it is unknown how this relationship relates to aspects of executive function performance in older adults. In the present study, we report a positive association between cardiorespiratory fitness and activation during dual-task processing within the ACC/SMA, thalamus/basal ganglia, right motor/somatosensory cortex and MFG, and left somatosensory cortex. Furthermore, ACC/SMA brain activation mediated the association between cardiorespiratory fitness and dual-task performance. Although the overall relationship between cardiorespiratory fitness and dual-task performance did not remain significant after controlling for various covariates, the results suggest that cardiorespiratory fitness facilitates behaviorally relevant modulation of brain activation. The four ROIs are areas that support dual-task processing in older adults, thereby extending previous findings of enhancement of behavioral indicators of dual-task performance, an important aspect of executive control, in older adults (Hawkins et al., 1992).

The present study demonstrates that components of the cognitive control network elicited by the dual-task paradigm are sensitive to cardiorespiratory fitness in older adults. Executive functioning is supported by the cognitive control network, which includes the ACC/SMA, PFC, premotor cortices, and the posterior parietal cortex. This cognitive control network is evoked by various task demands (Koski and Paus, 2000; Cole and Schneider, 2007; Niendam et al., 2012) and is also present during rest (Margulies et al., 2007). Here, higher cardiorespiratory fitness was related to greater activation in the dual-task condition relative to the single-task condition within the ACC/SMA, thalamus/basal ganglia, right motor/somatosensory cortex and MFG, and left somatosensory cortex, which overlap with areas within the cognitive control network. Of these brain regions where activation was related to cardiorespiratory fitness, only the ACC/SMA was positively associated with dual-task performance. Dosenbach et al. (2006) described the dorsal ACC and insula as core cognitive control regions because activity within those regions was sustained across a variety of task conditions. Other studies also connect the ACC to cognitive control or executive functions, such as multi-tasking (Medeiros-Ward et al., 2015), inhibition (Garavan et al., 2002), conflict monitoring (Kerns et al., 2004), error detection (Brown and Braver, 2005), and response selection (Paus et al., 1993; Milham et al., 2001). These studies provide evidence that the ACC/SMA may serve a unique role within the cognitive control network and dual-task processing.

We are not the first to suggest that the structure and function of the ACC is specifically sensitive to cardiorespiratory fitness in older adults. A number of longitudinal studies have demonstrated that the ACC exhibits a preferential change in both structure and function across time associated with exercise training. For example, older adults who participated in a walking program, 3 days per week for 6 months, had a significant increase in dorsal ACC/SMA regional brain volume, compared to a stretching and toning group (Colcombe et al., 2006). Additionally, Chapman et al. (2013) reported that 3 months of physical training exercise resulted in increased blood flow within the rostral ACC compared to a wait-list control group. Lastly, 4 months of exercise was associated with greater functional connectivity between the rostral ACC and the hippocampus, relative to a non-exercise group (Burdette et al., 2010). Despite the differences in the region of the ACC impacted by exercise, together these studies support that ACC is responsive to cardiorespiratory fitness and exercise interventions.

Cross-sectional studies have also linked ACC brain structure and function to cardiorespiratory fitness in older adults. One study demonstrated that fractional anisotropy, a measure of cerebral white matter integrity, within the middle cingulate cortex was positively associated with cardiorespiratory fitness in older adults (Marks et al., 2011). Additionally, older adults with higher and lower cardiorespiratory fitness differentially recruited the ACC during a selective attention task (Colcombe et al., 2004). Specifically, higher fit older adults exhibited reduced ACC activation and greater frontal-parietal activation compared to the lower fit older adults, which was interpreted as more effective attentional recruitment and less conflict compared to the lower fit older adults (Colcombe et al., 2004). In contrast, our data suggest that greater ACC activation during dual-task processing was associated with higher cardiorespiratory fitness and better dual-task performance. The different relationship between ACC activation and performance in these two studies may be a result of the nature of the tasks that subjects were performing – selective attention (i.e., focusing attention on a subset of stimuli in the display) in the Colcombe et al. (2004) study and dividing attention across the display, and multiple tasks, in the present study. Collectively, these studies provide additional evidence that the ACC region is sensitive to cardiorespiratory fitness. It may be that cardiorespiratory fitness alters the dynamic range of neural recruitment to improve neural efficiency and cognitive performance specific to the cognitive demands encountered in different tasks.

Our results extend the idea that the ACC/SMA region is particularly sensitive to cardiorespiratory fitness and important in executing cognitive control to manage tasks performed concurrently. We demonstrate that cardiorespiratory fitness related ACC/SMA activation serves to promote better cognitive performance by modulating cognitive control required during dual-task performance. A variety of dual-task paradigms elicited PFC and parietal activation during dual-task processing which was associated with dual-task performance. Specific PFC areas included the dorsal lateral prefrontal cortex (DLPFC) and ACC/SMA (D’Esposito et al., 1995; Adcock et al., 2000; Bunge et al., 2000; Szameitat et al., 2002; Schubert and Szameitat, 2003; Erickson et al., 2005). Whereas previous studies investigating dual-task brain function mainly focused on the DLPFC, converging evidence suggest that the ACC/SMA is associated with cardiorespiratory fitness, dual-task performance, and cognitive control. Within our dual-task paradigm greater ACC/SMA activation may be related to at least two aspects of dual-task performance, the maintenance and coordination of rules for the two distinct tasks, and mapping of the task-related stimuli to the appropriate motor responses (Paus et al., 1993, 1998; Picard and Strick, 1996). Performing simultaneous or demanding tasks elicit ACC recruitment, which may be associated with the increased possibility of error and conflict (Carter et al., 1998; Kerns et al., 2004). Greater ACC activation may serve to monitor processing and performance to modulate cognitive control by promoting active maintenance and coordination of multiple tasks. Consequently, our results suggest that the brain activation in ACC/SMA during dual-task processing mediated the relationship between cardiorespiratory fitness and dual-task performance by promoting greater cognitive control and improved performance monitoring as well as faster motor responses.

Whereas our study is the first to demonstrate that ACC/SMA brain activation mediated the association between cardiorespiratory fitness and dual-task performance, it must be viewed in the context of some limitations. First, it is important to note that fMRI detects changes in blood flow as an indirect measure of neuronal activity to measure brain activation. Differences in brain activation could be interpreted to reflect blood flow differences associated with cardiorespiratory fitness level rather than differences in neuronal function. However, if that were true, increased cardiorespiratory fitness would likely result in a global, non-specific increase in task-related brain activation. The dual-task processing results challenge such an idea by describing that cardiorespiratory fitness was associated with specific regional differences in task-related brain function. Additionally, we acknowledge while our sample of older adults is healthy, they are low active. Based on the American College of Sports Medicine the cardiorespiratory fitness level of our sample mean (21.26 ml/kg/min) is below the 50th age and sex predicted percentile (American College of Sports Medicine et al., 2010). Hence, we may be underestimating the associations with cardiorespiratory fitness and in future studies it would be beneficial to include a larger range of cardiorespiratory fitness levels. The cross-sectional nature of our study limits the casual conclusions that can be drawn, highlighting the importance of longitudinal randomized control trials. Furthermore, future research is needed to understand the neurobiological mechanisms by which cardiorespiratory fitness is associated with brain activation in older adults. Animal models have demonstrated that exercise promotes neurogenesis (van Praag et al., 2005), angiogenesis (Ding et al., 2006a), synaptic plasticity coupled with increased production of growth factors (e.g., brain-derived neurotrophic factor, insulin-like growth factor; Ding et al., 2006b; Marlatt et al., 2012), and reduced inflammation (da Silva et al., 2013). The extent to which these neurobiological mechanisms account for the positive effects of cardiorespiratory fitness on the aging human brain requires future exploration.

Our findings suggest that brain activation during dual-task processing is associated with individual differences in cardiorespiratory fitness and is directly relevant to dual-task performance in older adults. In particular, we report that higher cardiorespiratory fitness is associated with greater ACC/SMA brain activation, better cognitive control and more specifically, higher levels of dual-task processing. Our work is directly applicable to the growing older adult population, in that, an active lifestyle that supports cardiorespiratory fitness may be an effective and accessible way to combat the negative aspects of the cognitive aging process. Consequently, older adults with greater cardiorespiratory fitness may be able to maintain their independence and continue to positively contribute to society.

## Conflict of Interest Statement

The authors declare that the research was conducted in the absence of any commercial or financial relationships that could be construed as a potential conflict of interest.

## Footnotes

1 www.fmrib.ox.ac.uk/fsl

2 http://fsl.fmrib.ox.ac.uk/fsl/fslwiki/FEAT

3 www.quantpsy.org.
